# Supplementary material for: Pyruvate Dehydrogenase Kinase 1 inhibition mediated oxidative phosphorylation enhancement in cartilage promotes osteoarthritis progression
Source: BMC Musculoskelet Disord. 2023 Jul 20;24:597. doi: 10.1186/s12891-023-06585-6 (PMC10357736; doi:10.1186/s12891-023-06585-6)
Supplement: Supplementary file 1 — Additional file 1. [file 12891_2023_6585_MOESM1_ESM.zip › BP.Gsea.1653623667859/GOBP_REGULATION_OF_FATTY_ACID_BETA_OXIDATION.html]

Details for gene set GOBP\_REGULATION\_OF\_FATTY\_ACID\_BETA\_OXIDATION[GSEA]

|  || Dataset | 55457\_collapsed\_to\_symbols.cls.cls #OA\_versus\_normal.cls.cls #OA\_versus\_normal\_repos |
| Phenotype | cls.cls#OA\_versus\_normal\_repos |
| Upregulated in class | normal |
| GeneSet | GOBP\_REGULATION\_OF\_FATTY\_ACID\_BETA\_OXIDATION |
| Enrichment Score (ES) | -0.6172875 |
| Normalized Enrichment Score (NES) | -1.7437762 |
| Nominal p-value | 0.009009009 |
| FDR q-value | 0.11744472 |
| FWER p-Value | 0.999 |
Table: GSEA Results Summary

  

Fig 1: Enrichment plot: GOBP\_REGULATION\_OF\_FATTY\_ACID\_BETA\_OXIDATION      
 Profile of the Running ES Score & Positions of GeneSet Members on the Rank Ordered List

  

| SYMBOL | TITLE | RANK IN GENE LIST | RANK METRIC SCORE | RUNNING ES | CORE ENRICHMENT || 1 | AKT1 | AKT serine/threonine kinase 1 [Source:HGNC Symbol;Acc:HGNC:391] | 1192 | 0.478 | -0.0166 | No |
| 2 | ABCD2 | ATP binding cassette subfamily D member 2 [Source:HGNC Symbol;Acc:HGNC:66] | 3031 | 0.246 | -0.1201 | No |
| 3 | MLYCD | malonyl-CoA decarboxylase [Source:HGNC Symbol;Acc:HGNC:7150] | 4852 | 0.083 | -0.2481 | No |
| 4 | FABP1 | fatty acid binding protein 1 [Source:HGNC Symbol;Acc:HGNC:3555] | 7604 | -0.127 | -0.4414 | No |
| 5 | ABCB11 | ATP binding cassette subfamily B member 11 [Source:HGNC Symbol;Acc:HGNC:42] | 7825 | -0.143 | -0.4358 | No |
| 6 | CPT1A | carnitine palmitoyltransferase 1A [Source:HGNC Symbol;Acc:HGNC:2328] | 8046 | -0.159 | -0.4276 | No |
| 7 | ABCD1 | ATP binding cassette subfamily D member 1 [Source:HGNC Symbol;Acc:HGNC:61] | 8930 | -0.226 | -0.4602 | No |
| 8 | ACACB | acetyl-CoA carboxylase beta [Source:HGNC Symbol;Acc:HGNC:85] | 10955 | -0.425 | -0.5498 | Yes |
| 9 | PPARA | peroxisome proliferator activated receptor alpha [Source:HGNC Symbol;Acc:HGNC:9232] | 11110 | -0.445 | -0.4911 | Yes |
| 10 | CNR1 | cannabinoid receptor 1 [Source:HGNC Symbol;Acc:HGNC:2159] | 11181 | -0.454 | -0.4244 | Yes |
| 11 | IRS2 | insulin receptor substrate 2 [Source:HGNC Symbol;Acc:HGNC:6126] | 11390 | -0.485 | -0.3636 | Yes |
| 12 | TWIST1 | twist family bHLH transcription factor 1 [Source:HGNC Symbol;Acc:HGNC:12428] | 11696 | -0.531 | -0.3029 | Yes |
| 13 | LONP2 | "lon peptidase 2, peroxisomal [Source:HGNC Symbol;Acc:HGNC:20598]" | 11699 | -0.532 | -0.2185 | Yes |
| 14 | IRS1 | insulin receptor substrate 1 [Source:HGNC Symbol;Acc:HGNC:6125] | 11841 | -0.557 | -0.1408 | Yes |
| 15 | AKT2 | AKT serine/threonine kinase 2 [Source:HGNC Symbol;Acc:HGNC:392] | 12896 | -1.404 | 0.0003 | Yes |
Table: GSEA details [plain text format]

  

Fig 2: GOBP\_REGULATION\_OF\_FATTY\_ACID\_BETA\_OXIDATION      
 Blue-Pink O' Gram in the Space of the Analyzed GeneSet

  

Fig 3: GOBP\_REGULATION\_OF\_FATTY\_ACID\_BETA\_OXIDATION: Random ES distribution      
 Gene set null distribution of ES for **GOBP\_REGULATION\_OF\_FATTY\_ACID\_BETA\_OXIDATION**
